# Electron‐Beam Excited Conductive Atomic Force Microscopy for Back Contact Free, Wafer‐Scale and In‐Line Compatible Electrical Characterization of 2D Materials

**DOI:** 10.1002/advs.202505113

**Published:** 2025-08-27

**Authors:** Md Ashiqur Rahman Laskar, Sakib Ahmed, Pinakapani Tummala, Alessandro Molle, Alessio Lamperti, Renee Sailus, Youssry Y Botros, Milan Pesic, Rob Davenport, Ondřej Novotný, Jan Neuman, Fabrizio Toia, Ivan Sanchez Esqueda, Seth Ariel Tongay, Umberto Celano

**Affiliations:** ^1^ School of Electrical, Computer, and Energy Engineering Arizona State University Tempe AZ 85281 USA; ^2^ CNR IMM Unit of Agrate Brianza, via C. Olivetti 2 Agrate Brianza 20864 Italy; ^3^ School for Matter, Transport and Energy Engineering Arizona State University Tempe AZ 85287 USA; ^4^ Applied Materials Inc. Santa Clara CA 95054 USA; ^5^ NenoVision s. r. o. Brno 61200 Czech Republic; ^6^ STMicroelectronics Via C. Olivetti 2 Agrate Brianza 20864 Italy

**Keywords:** 2D materials, conductive AFM, electrical characterization, in‐line metrology, MoS_2_, wafer compatible

## Abstract

Electrical atomic force microscopies (AFMs) have emerged as leading metrology techniques for evaluating the quality of 2D materials. Their advantages include high‐resolution electrical mapping, non‐destructive measurement, and the ability to probe nanoscale defects and transport properties. Conductive AFM (C‐AFM) has been particularly instrumental, enabling the direct observation of individual vacancies and vacancy clusters, voids, wrinkles, and cracks. Despite this incredible versatility, C‐AFM remains a two‐probes method, thus it is limited by the need for physical back‐contact. Creating this back contact is complex and time‐consuming. More importantly, this requirement prevents C‐AFM from being a viable in‐line metrology technique. Here, it is demonstrated that a low‐energy e‐beam impinging on the sample surface can be used to perform C‐AFM, in a new configuration that is electron‐beam (e‐beam) excited conductive atomic force microscopy (EBC‐AFM). This approach enables comparable results to classic C‐AFM sensitivity, while unlocking applications that were not previously possible. After introducing the experimental setup, the main parameters associated with the e‐beam and their impact on the C‐AFM measurement are studied. Finally, using several 2D materials as testbeds, the competitive electrical mapping capabilities of EBC‐AFM for defect assessment are demonstrated. Furthermore, this technique overcomes limitations for studying isolated flakes and enables wafer‐scale characterization of 2D materials.

## Graphical abstract

Electron‐beam excited conductive atomic force microscopy is demonstrated. Here, a low‐energy e‐beam impinging on the sample surface can be used to perform C‐AFM in a new configuration that enables comparable results to classic C‐AFM sensitivity, while unlocking applications previously not possible in terms of large‐area analysis and wafer‐scale metrology.

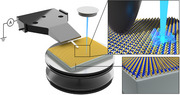

## 1 Introduction

2D materials have emerged as a transformative class of materials with remarkable potential for revolutionizing electronics. These materials are atomically thin or only a few atoms thick, which exhibit extraordinary electronic properties that set them apart from conventional bulk materials. Since the discovery of graphene in 2004, numerous other 2D materials have been introduced till the present day, where each of them offers unique characteristics such as semiconductor, metal, semi‐metal, insulator, etc. that are suitable for diverse applications.^[^ ^1^, ^2^, ^3^ ^]^ Transition metal dichalcogenides (TMDs), e.g., molybdenum disulfide (MoS_2_), are semiconducting 2D materials with a tunable bandgap, making them suitable for use in field‐effect transistors (FETs) and photonics applications.^[^ ^4^, ^5^, ^6^ ^]^ Importantly, 2D‐TMDs materials are playing a crucial role in addressing the limitations of silicon‐based technologies, providing new possibilities for transistor design and performance, including the development of ultrathin channel transistors and emerging 3D integration.^[^ ^7^, ^8^ ^]^ As research advances, the scalability, stability, and integration of 2D materials into electronic devices are further unlocking their potential for commercial application and high volume manufacturing (HVM). However, such integration in HVM necessarily requires maintaining high quality, uniformity, and defect control of 2D materials during each step of synthesis/direct growth, transfer, integration, and device fabrication, etc.^[^ ^9^ ^]^

At this point, an important pillar for the advancement of 2D materials hinges on developing robust and reliable metrology techniques for electrical characterization, particularly those suitable for large‐scale production. Traditional methods for characterizing the electrical properties of materials often present significant limitations when applied to 2D materials, which are inherently delicate and have unique properties. The advent of 2D materials in chip manufacturing brings new opportunities for devices, as well as new challenges for large‐area synthesis, integration, and metrology.^[^ ^10^ ^]^ A key challenge is the need for techniques that are compatible with wafer‐scale fabrication and in‐line processing, as these are essential for translating laboratory discoveries into commercial products. Scanning probe microscopies (SPM), including scanning tunneling microscopy (STM) and AFM, have demonstrated the potential to sense intrinsic and extrinsic material quality and efficient mapping of structural, electrical, and optical properties.^[^ ^11^, ^12^ ^]^ Among these, electrical AFMs have become a workhorse to characterize the electrical properties of 2D materials due to the capability of identifying local defects such as vacancies, adatoms, substitutional impurities, grain boundaries (GBs), and to qualify their physical properties, as well as carrier donor/acceptor and scattering, trap, and recombination centers in many 2D materials.^[^ ^13^, ^14^, ^15^, ^16^, ^17^, ^18^ ^]^

**Figure 1:**
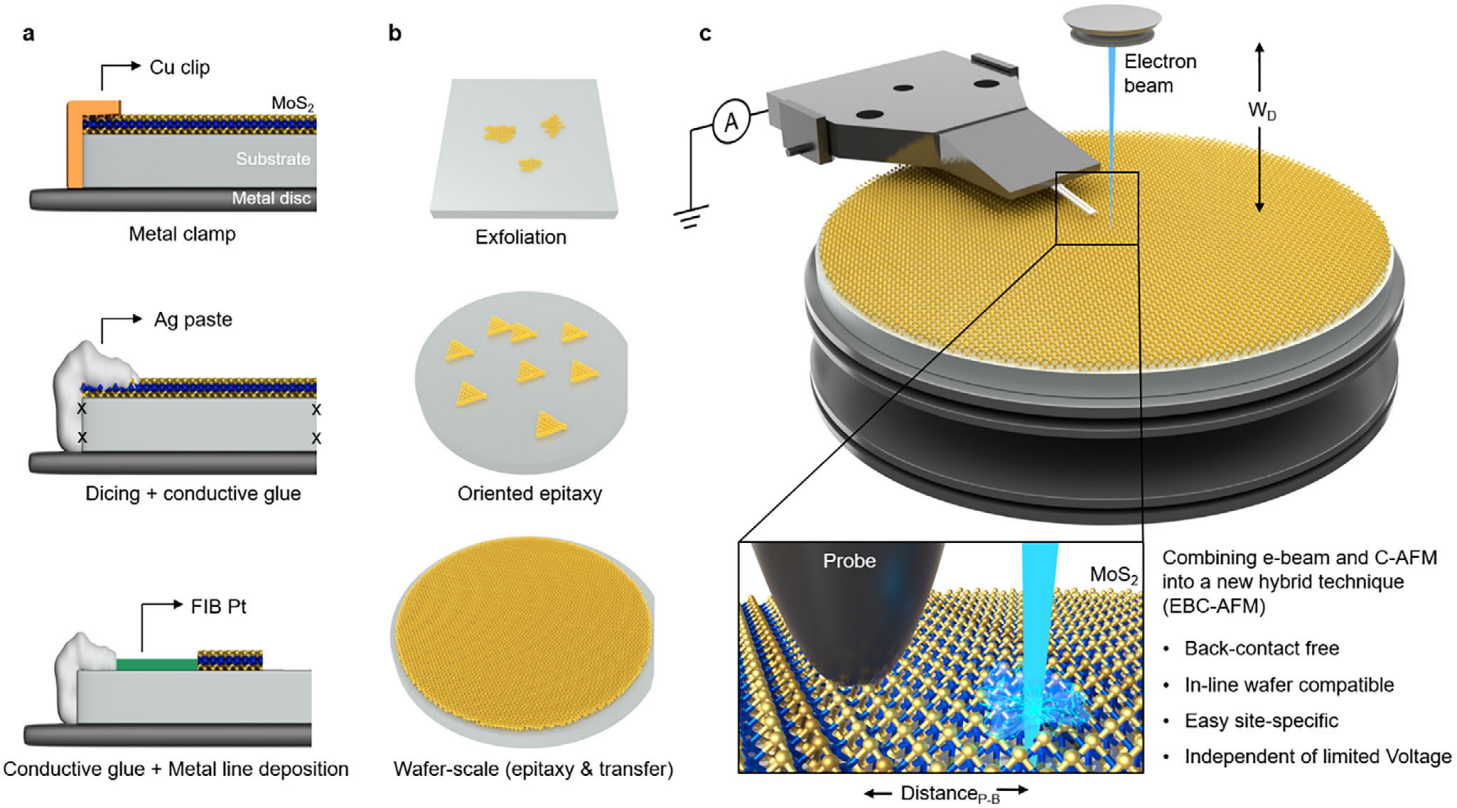
a) Schematics showing different existing methods of back‐contact creation on 2D material sample for conventional C‐AFM. b) Schematic of 2D material on insulator substrate or layer obtained by mechanical exfoliation, epitaxy growth, and wafer‐scale CVD growth or post growth wafer‐scale transfer. c) Schematic of EBC‐ AFM configuration with a 2D material wafer sample. Here, the wafer substrate is an insulator, and W_D_ means the working distance (few mm), indicating the distance between the SEM bottom column and the sample surface. Distance_P‐B_ indicates the physical distance (tens to hundreds of µm) between the AFM probe and the electron spot beam on the sample.

However, often based on two‐probes implementation, conventional electrical AFMs require the creation of a back‐contact on the sample,^[^ ^19^, ^20^ ^]^ which poses significant challenges for thin films, non‐continuous films, or island‐like 2D material samples. A classic example is represented by conductive atomic force microscopy, which has shown STM‐comparable results in many applications using 2D materials but is plagued by the need for small coupon samples and electrical back‐contact.^[^ ^21^ ^]^ Existing methods for back‐contact creation, including the use of metallic clips, conductive glues, or focused ion beam (FIB) deposition,^[^ ^22^, ^23^ ^]^ often lead to issues such as sample damage, contamination, high contact resistance, or complex and time‐consuming preparation procedures (**Figure** **1**a). Beyond these limitations hinder the efficient characterization of 2D materials, particularly for in‐line and wafer‐scale measurements.

Here, we introduce ‐ EBC‐AFM as a novel technique designed for the electrical characterization of 2D materials that eliminates the need for physical back‐contact. EBC‐AFM overcomes these limitations by utilizing an e‐beam in the vicinity of the scanning probe to create a movable electrical contact acting as a pathway for current, thus removing the need for physical back‐contact and, as a result, the need for the use of small coupons. We start by describing the technical implementation of EBC‐AFM. Here, we demonstrate how the presence of e‐beam provides control over C‐AFM measurements through parameters such as beam current and acceleration voltage, and can be implemented using commercially available AFM‐in‐SEM modules. We continue with the demonstration of EBC‐AFM functionality in material systems completely isolated from the AFM stage, e.g., isolated exfoliated flakes, and as‐grown MoS_2_ on sapphire. Finally, we compare our method for the characterization of synthetic MoS_2_ layers grown on different substrates, including sapphire, SiO_2_/Si, and directly grown on a fully patterned integrated circuit (IC) die fully compatible with large‐area wafer scale growth and metrology.

## 2 Discussion

In traditional C‐AFM experiments, creation of back‐contact is compulsory, and there are very few ways to do that. For example, as shown in Figure 1a, the easiest method includes the use of metallic clips (or a clamp) such as Al or Cu, however, this solution is often inconvenient for very thin films or monolayers 2D material samples because the clamp easily penetrates the layer of interest, causing a poor electrical contact. Therefore, it is common to apply a small amount of InGa eutectic followed by conductive glue (e.g., fast‐drying silver paste) on the surface to create a large ohmic contact. This requires additional sample preparation steps, including dicing a coupon sample, sacrificing a small area for the Ag paste, applying thermal annealing for the evaporation of solvents in the glue, etc. In addition, this method is not suitable for non‐continuous films or isolated islands, as it does not guarantee electrical connection in these cases. Alternatively, conductive tapes have similar problems and often result in high contact resistance (several MΩ) because of the adhesion issues with 2D materials. As indicated in Figure 1b, islands like 2D material samples, for instance, non‐continuous MoS_2_ film obtained by mechanical exfoliation or epitaxial growth, make it very difficult to create the necessary back‐contact. In these cases, one possible solution is represented by the physical creation of a metallic line (platinum or gold) deposited by a focused ion beam or thermal evaporation with shadow masking, which are solutions that generally add complexity and time‐consuming for the sample preparation. As such, it is no surprise that the community considers electrical tip‐based techniques slow, low throughput, limited in their fields of view, and therefore not economical for in‐line and high‐volume wafer scale characterization.

However, despite the additional complexity added in the sample preparation, the role of back‐contact in C‐AFM is mainly the one of charge injections. Therefore, here we opted to replace it with an electron beam to serve the same function. Figure 1c represents the schematic of the proposed EBC‐AFM setup where a conductive probe is scanning in contact‐mode while the electron beam (spot mode) of a scanning electron microscope (SEM) is impinging on the sample surface in proximity of the scanning area. The result is that the incident beam supplies a substantial amount of electrons which spread over the sample volume, causing localized charging and a net negative potential, ‐V_s_ on the sample surface.^[^ ^24^, ^25^ ^]^ Since the AFM probe is conductive and connected to the ground during the raster scans, the electrons flow toward the ground through a current measuring amplifier unit effectively performing a C‐AFM measurement in the absence of any traditional back‐contacts. A comparison between a traditional C‐AFM setup and the EBC‐AFM is reported in Figure S1 (Supporting Information). In this work, we implemented the EBC‐AFM using a commercial AFM‐in‐SEM module (Litescope 2.0) in combination with various SEM systems such as Nova 200 NanoLab and Helios 5 UX (ThermoScientific). The probes utilized are Pt coated self‐sensing (piezo‐resistive) probes operating in contact mode, and conventional Pt coated probes.

**Figure 2:**
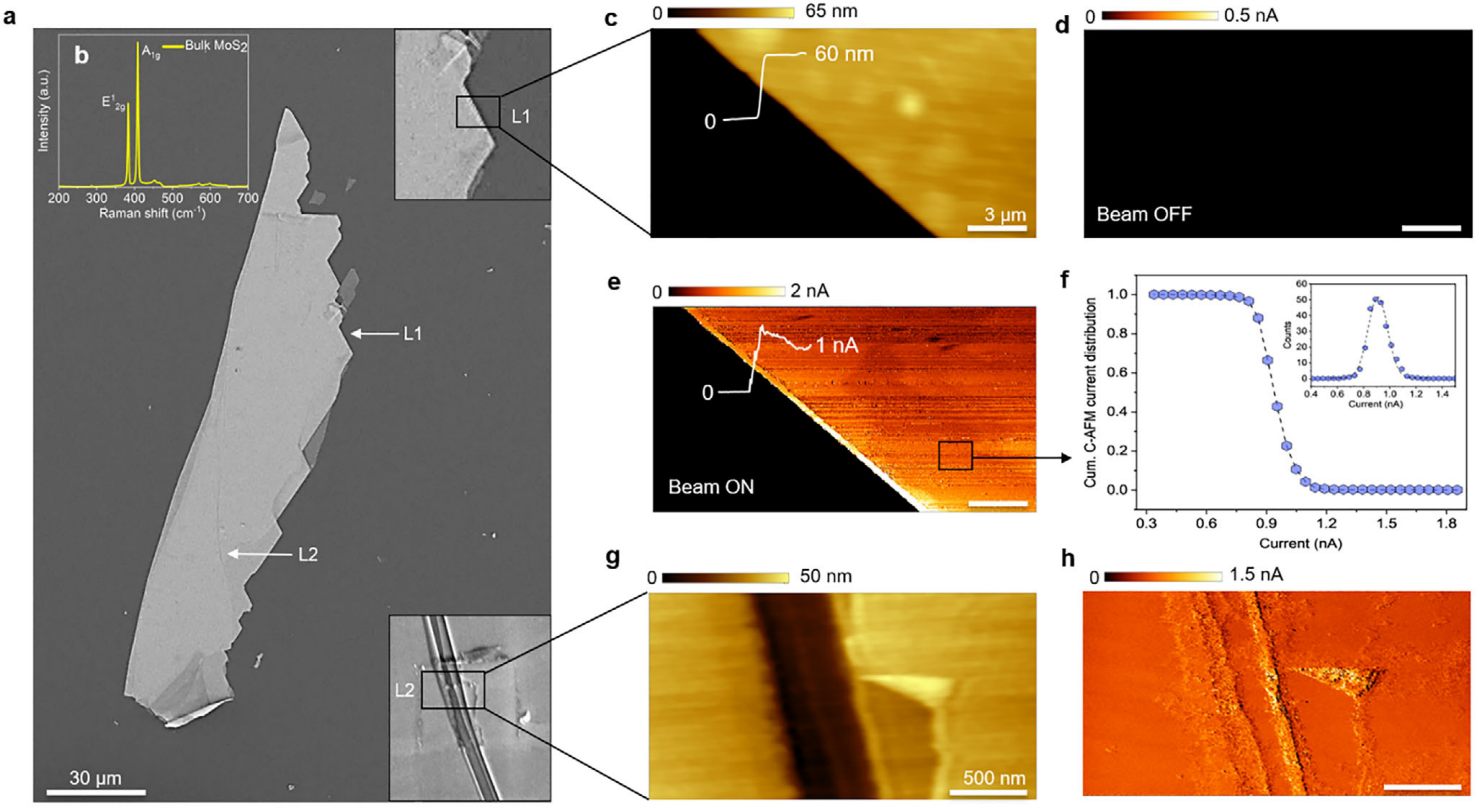
a) SEM image of exfoliated MoS_2_ flake (bulk) on SiO_2_(300 nm)/Si substrate. Scale bar is 30 µm. L1 and L2 are two different locations on the flake that have been shown in zoomed view as inset images. b) Raman spectra of the corresponding MoS_2_ flake taken with a 488 nm laser. The in‐plane and out‐of‐plane vibrational peaks are indicated by E^1^_2g_ and A_1g,_ respectively. c) Large area AFM topography of MoS_2_ at location L1. Scale bar is 3 µm. d,e) C‐AFM image of corresponding location L1 keeping the electron beam OFF and ON conditions, respectively. During OFF condition, maximum ±10 V is applied between the probe and chuck. In contrast, during ON condition (EBC‐AFM), the beam shines 50 µm away from the scanning probe in spot mode at 5 kV acceleration voltage (V_acc_) and 0.4 nA beam current (I_beam_). No voltage is applied between the probe and chuck. f) Cumulative current distribution plot of the designated box area. Inset: Number of counts versus C‐AFM current. g,h) Small area AFM topography and C‐AFM image of MoS_2,_ respectively at location L2 obtained by EBC‐AFM with the same beam parameter. The scale bar is 500 nm.

For the primary validation of the EBC‐AFM, we start by considering an isolated exfoliated MoS_2_ flake transferred on a SiO_2_/Si substrate as shown in **Figure** **2**a. For extra precautions, we mount the whole sample on top of an insulating tape to ensure it is completely electrically isolated from the chuck (Figure S2, Supporting Information). The sample considered here is a mechanically exfoliated MoS_2_ sample with characteristics of a bulk sample as revealed by the Raman spectra in Figure 2b;^[^ ^26^, ^27^ ^]^ the in‐plane (E^1^_2 g_) vibrational mode is at red shifted (382 cm^−1^) and out‐of‐plane (A_1g_) mode is at blue shifted (408.5 cm^−1^) peak positions. The flake thickness is 60 nm as extracted from the morphology of AFM (Figure 2c). Figure 2d shows the attempt to measure this flake with conventional C‐AFM by applying the maximum sample bias (i.e., ±10 V) in the location L1 (18 × 9 µm^2^), with no visible current contrast in the secondary channel. In contrast, in Figure 2e the tip is grounded, and the e‐beam is ON impinging a few microns away from the location of the scan. It is clear that a uniform C‐AFM contrast can be obtained in this contact‐less configuration by replacing the back‐contact with the e‐beam. As expected for this type of sample and at this dimension, the current channel presents a uniform distribution of conductive pixels. This is reported in Figure 2f where we present the cumulative distribution of the current extracted from the C‐AFM map, with values showing a Gaussian distribution centered ≈0.9 nA. Figure 2g,h shows a smaller scan area (2 × 1 µm^2^) at location L2, here increasing the resolution of the analysis with a comparable result. The most noticeable features in the AFM morphology here can also be clearly detected in the current channel.

**Figure 3:**
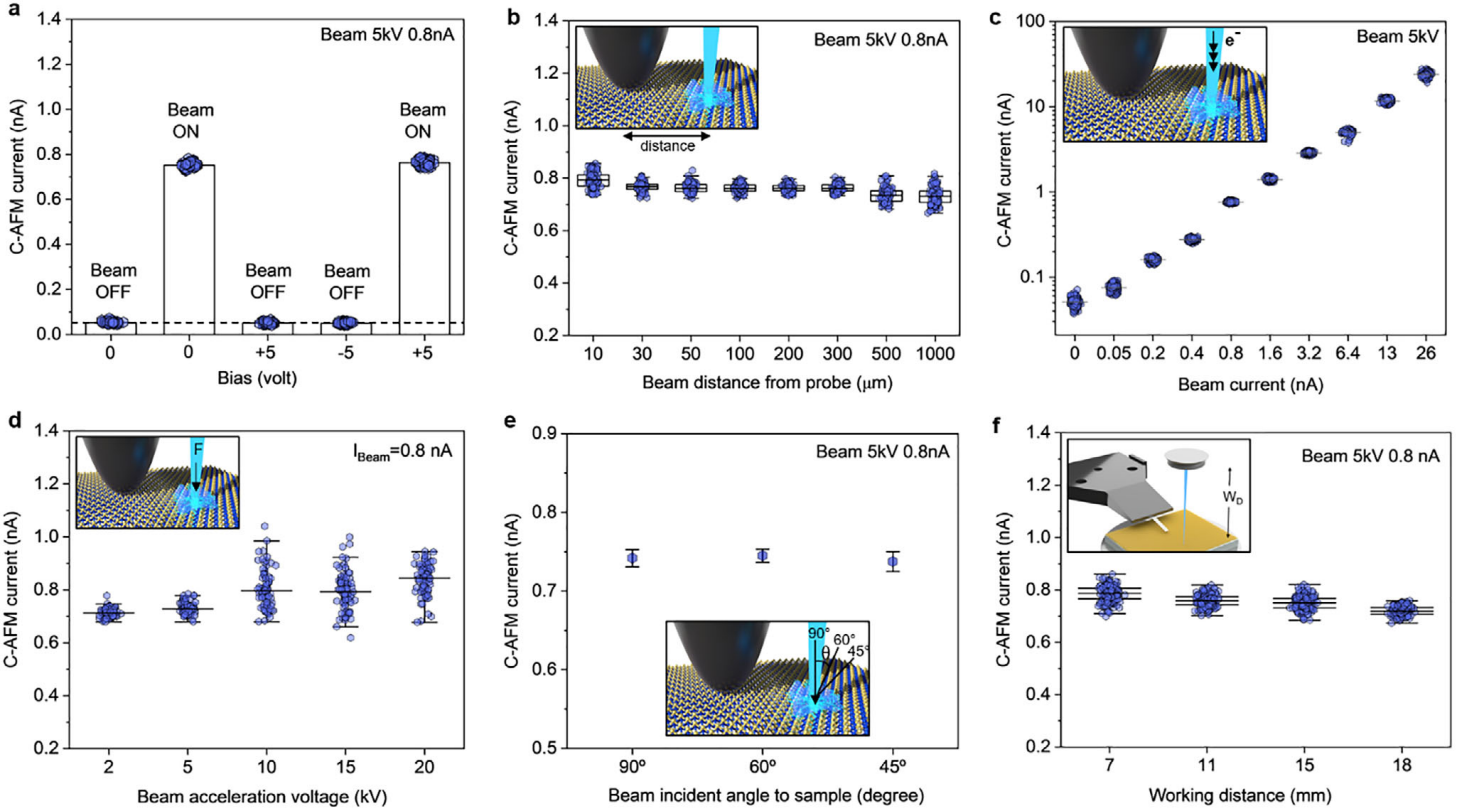
Various C‐AFM data collected through point contact based current versus time (I‐T) spectroscopies on a 3‐5 monolayers MoS_2_/SiO_2_/Si sample by EBC‐AFM technique: a) C‐AFM current versus applied bias plot at electron beam ON/OFF condition in spot mode. When the beam is ON, it shines at 5 kV acceleration voltage, 0.8 nA beam current, and beam incident angle (**∠**i) 90°. The dash line indicates the noise floor of the trans‐impedance amplifier of the Litescope C‐AFM sensor. b) Plot of C‐AFM current versus electron beam distance from the probe while illuminating e‐beam at V_acc_=5 kV, I_beam_=0.8 nA, **∠**i=90°. c) C‐AFM current versus beam current plot when the e‐beam is at distance_P‐B_=50 µm and V_acc_=5 kV, and **∠**i=90°. d) Plot of C‐AFM current versus different beam acceleration voltages when I_beam_=0.8 nA, distance_P‐B_=50 µm, and **∠**i=90°. e) Plot showing the C‐AFM current with respect to three different beam incident angles to sample surface when V_acc_=5 kV, I_beam_=0.8 nA, and distance_P‐B_=50 µm. Working distance, W_D_ is 10 mm for all the data shown here in figures (a–e). f) C‐AFM current versus working distance while illuminating e‐beam at V_acc_=5 kV, I_beam_=0.8 nA, **∠**i=90°, and distance_P‐B_=50 µm.

While Figure 2 indicates the feasibility of the EBC‐AFM approach, here we report on a systematic study to understand the impact of the e‐beam parameters on the C‐AFM readouts. To this end, we focus on a 3‐5 monolayers (MLs) MoS_2_/SiO_2_/Si sample grown by ambient pressure chemical vapor deposition (AP‐CVD), and explore the parameter space offered by the modulation of stage bias, electron beam distance, beam current, acceleration voltage, and beam angle, etc. For each of these parameters we report on their impact on the C‐AFM current, measured by means of static contact based current versus time (I‐T) spectroscopy. The aim is to minimize all possible motion‐induced effects, typical of a scanning probe, while focusing on the best e‐beam conditions to maximize the signal‐to‐noise (S/N) ratio in our setup. For the sake of the statistics, we repeated each measurement in three distinct locations on the sample and show the results in **Figure** **3**.

As a first sanity check, we demonstrate that the MoS_2_ layer is isolated from the chuck, for example, the bar plot in Figure 3a shows how there is no C‐AFM current when we apply ±5 V to the sample stage (with e‐beam OFF). This confirms how the MoS_2_ is isolated, thanks to the SiO_2_ layer, additionally the whole sample is mounted by an insulating tape and there is no back‐contact. Note, a small amount of current ca. 50 pA is always visible in this configuration as indicated by the dash line, and it is the noise floor of our trans‐impedance amplifier, i.e., the C‐AFM sensor. In this way, the effect of the external bias (applied to the chuck) is neutralized as indicated by the dependence of the C‐AFM current only visible with electron beam illumination ON, and showing no change regardless of the applied bias. Figure 3b represents the C‐AFM current with respect to different beam distances from the conductive probe while keeping the beam acceleration voltage, V_acc_ = 5 kV and beam current, I_beam_ = 0.8 nA as constant. It is observed that an average current of 0.76 nA prevails when the electron beam impinges on MoS_2_ within the range of 30–300 µm distance from the probe. However, differences can be observed as a function of the probe location. For example, a higher average current of 0.79 nA is observed when the beam is only 10 µm away from the probe. Here, it is important to mention the rich physics of e‐beam matter interaction that includes the generation of secondary emitted (SE) and backscattered (BS) electrons escaping from the sample and encountering directly a closely located conductive probe. However, we see this effect to stabilize starting from ca. 30 µm from the probe location, thus we consider this distance as a threshold to avoid parasitic charge injection from SE and BS electrons into the probe. Besides, a certain minimum distance ensures to avoid any interference to the C‐AFM, which may be caused by the accumulated charges on surface (charging effect) near the beam illumination spot. While 30 µm of minimum distance between probe and e‐beam is considered safe to avoid parasitic charge injection into the probe, we notice a change in the acquired data when increasing this distance over 300 µm (Figure 3b). Here, a slightly lower average current of 0.73 nA is observed, for example, with the beam at 500 µm distance or more, we speculate that in this material system, this value is set by the intrinsic series resistance added by the resistivity of the MoS_2_ at such distances.^[^ ^28^ ^]^ This simple observation allows us to point out that the EBC‐AFM approach can easily overcome these resistivity‐induced contact limitations by moving the e‐beam to an optimal distance from the area under study.^[^ ^28^ ^]^

In addition, as indicated by Figure S3 (Supporting Information), it is important to note that the influence of escaping SE and BS electrons on C‐AFM measurement by direct encountering to the probe is negligible in such recommended distance_P‐B_ for the trivial height (H < 20 µm) of the scanning probe. It's because the trajectory of SE/BS electrons is dependent on cosine functions,^[^ ^29^, ^30^, ^31^ ^]^ which indicates that most of the electrons are emitted at angles close to normal to the sample surface, with the probability of emission decreasing as the angle deviates from the surface normal. Thus, the number of escaping SE/BS electrons becomes insignificant near the sample surface, hence near the tip of the conductive probe.

Figure 3c demonstrates the C‐AFM current level on the MoS_2_ sample as a function of applied electron beam current. I_beam_, often referred to as beam intensity in SEM, denotes the quantity of electrons in the e‐beam striking the sample per unit of time. Keeping the V_acc_ as constant at 5 kV, we vary the I_beam_ from 0 to 26 nA to observe the changes in C‐AFM current. The observation divulges that the current level rises almost linearly with the gradual increase of I_beam_ for a large range. For example, when applied I_beam_ = 0.8, 1.6, 3.2 nA, etc., the corresponding C‐AFM current shows an average of 0.76, 1.4, and 2.85 nA, respectively. Thus, it indicates our EBC‐AFM technique can provide a good control over the C‐AFM measurement as required for different types of samples. It is believed that the majority of measured C‐AFM current in EBC‐AFM method is actually contributed from the primary electrons of the applied beam although the unescaped SE/BS electrons on the sample surface might have some contribution depending on the sample type. In general, the C‐AFM current will not be the same as the incident I_beam_ due to electron losses through absorption and energy transfer. Otherwise, measured C‐AFM current can be higher than the I_beam_ when the sample has a high secondary electron emission yield and the unescaped SE/BS electrons contribute significantly. Next, we analyze the effect of beam acceleration voltage on C‐AFM measurement while keeping the I_beam_ = 0.8 nA as constant. The higher V_acc_ means that the electrons in the beam are given more kinetic energy, causing them to penetrate deeper into a sample with a higher force, resulting in a larger interaction volume and generating more SE/BS electrons. As plotted in Figure 3d, the C‐AFM current looks stable and confined at ≈0.72 nA for low V_acc_ of 2–5 kV range. However, an increase in average with a large scattered current value is observed for high V_acc_ in the range of 10–20 kV. As an example, the C‐AFM current at V_acc_ = 10 kV disperses in between 0.7 and 1 nA with an average current of 0.8 nA. We anticipate that such dispersion with an increased current level is a consequence of substantial unescaped SE electrons, surface charging effects, and direct encountering SE/BS electrons to the probe, etc. As shown in Figure S4 (Supporting Information), Monte Carlo simulation^[^ ^32^ ^]^ of electron trajectories illustrates that increasing the V_acc_ from 5 to 10 kV results in almost 3x higher interacting depth and width within the sample when the primary electrons (blue) strike and penetrate it. In EBC‐AFM, low V_acc_ (within 5 kV) is recommended since higher V_acc_ can cause not only elevated charging effects on the sample surface but also damage to the sample. Prior studies point out that high‐energy electron beam irradiation can introduce defects (e.g., atomic vacancies) in the 2D materials when the V_acc_ is usually larger than 50 kV.^[^ ^33^, ^34^, ^35^ ^]^ In case low V_acc_ causes any minor defect formation in the 2D material,^[^ ^36^ ^]^ it is noteworthy to mention that the C‐AFM scanning region is always kept tens to hundreds of µm away from the electron beam spot and the spot size is ˂ 50 nm in diameter. Therefore, there will be no impact on C‐AFM measurement of the scanned region. Moreover, we conduct a simple test to determine if the electron beam irradiation brings about any direct damage or structural changes to the MoS_2_ during the EBC‐AFM experiment. As presented in Figure S5 (Supporting Information), a 5 × 5 µm^2^ region on a MoS_2_ (3‐5 MLs) sample is continuously exposed to electron beam irradiation for 15 min at V_acc_ = 5 kV and I_beam_ = 1.6 nA in spot mode. We perform Raman spectroscopy and conventional C‐AFM with identical parameters exactly on the same exposed region before and after the beam irradiation. The comparison of Raman spectra discloses that there is no shift or changes in the characteristic vibrational peaks (E^1^_2 g_ and A_1g_) of MoS_2,_ indicating no structural damage to the MoS_2_. Besides, the C‐AFM data comparison also agrees with the Raman observation. It is to clarify that recommended low V_acc_ (≤5 kV) does not limit the characterization of 2D materials in terms of their thickness or number of monolayers. On the other hand, substrate‐trapped charge induced by the electron irradiation can generate undesired effects such as charge accumulation on the material and carrier scattering affecting the mobility in the region where the e‐beam is impinging.^[^ ^37^, ^38^ ^]^ However, our results suggest that the combined effect of a large perimeter of charge injection and the relatively long distance from the EBC‐AFM scanning area and the e‐beam landing point does not make a clear effect in our tip‐sample current measurement. Nonetheless, it is suggested to use lower V_acc_ and I_beam_ depending on the material system for avoiding any parasitic signal on the conductive probe. In fact, choosing an environmental SEM or low vacuum inside the e‐beam system can help minimizing the overall charging effect by neutralizing some of the redundant electrons on the sample surface.^[^ ^39^, ^40^, ^41^ ^]^

Thereafter, we investigate the impacts of electron beam incident angle and the working distance on the EBC‐AFM experiment independently, keeping all other parameters constant. As shown in Figure S6 (Supporting Information), we tilt the sample at different angles with respect to the e‐beam and carry out the C‐AFM spectroscopies. The measured current level almost remains the same for all the beam incident angles of **∠**i = 90°, 60°, and 45° as depicted in Figure 3e. That means our technique is independent of **∠**i or any sample tilting limitations, allowing it more flexibility during the experiment. On the other hand, when the beam incident angle deviates from the perpendicular position, the escape depth (d) of SE electrons is reduced by d×cosθ (θ is the angle deviation from the perpendicular position of the beam due to sample tilt), resulting in an increase of SE electron emission from the sample.^[^ ^42^, ^43^ ^]^ Since we do not observe any visible increase in C‐AFM current level at different angles, it indicates the conductive probe does not collect escaping SE electrons, and thus, the EBC‐AFM technique is free from SE electron interference if using appropriate low V_acc_ and recommended beam distance as discussed previously. Figure 3f displays the C‐AFM current with regard to the working distance changes from low to high. As the W_D_ increases from 7 to 18 mm, a tiny decrease of 0.06 nA is observed in the average current value. It is possible that the electron beam diverges more with higher W_D_, resulting in a larger spot size on the sample and less SE electrons generation in overall. As previously discussed, the escaped SE electrons usually do not contribute to the C‐AFM current, however, the generated and unescaped SE electrons that travel on the sample surface through MoS_2_ can have a slight contribution. Therefore, the unescaped SE electrons will also be less as generated when W_D_ = 18 mm in compared to 7 mm. This might be the reason for the tiny decrease in average C‐AFM current as mentioned above.

Additionally, we repeated a few of the above experiments related to beam distance, beam current and incident angle on a conductive Cu wire as illustrated in Figure S7 (Supporting Information). Using V_acc_ = 5 kV, I_beam_ = 0.4 nA and W_D_ = 10 mm, we have observed that all the data are in accordance with our previous findings for the MoS_2_ sample. The slight decrease in C‐AFM current level for the MoS_2_ sample when distance_P‐B_> 500 µm is no longer visible here in case of Cu wire, obviously due to its negligible series resistance. On top of this, we also perform a complete C‐AFM scan on the Cu wire as shown in Figure S8 (Supporting Information). It is clear that the current map obtained by EBC‐AFM technique provides interesting details from the Cu surface and it follows the topography as well. As observed in both Figures S6 and S7 (Supporting Information), the higher current level in the EBC‐AFM compared to the applied I_beam_ can be explained in terms of SE electrons. Indeed, Cu exhibits a higher secondary electron emission yield due to its metallic properties allowing for easier electron excitation and transportation in comparison to the 2D materials. Essentially, more SE electrons are generated in Cu under e‐beam illumination and unescaped portion contributes to the overall carrier concentration increase that flows through the Cu wire.

**Figure 4:**
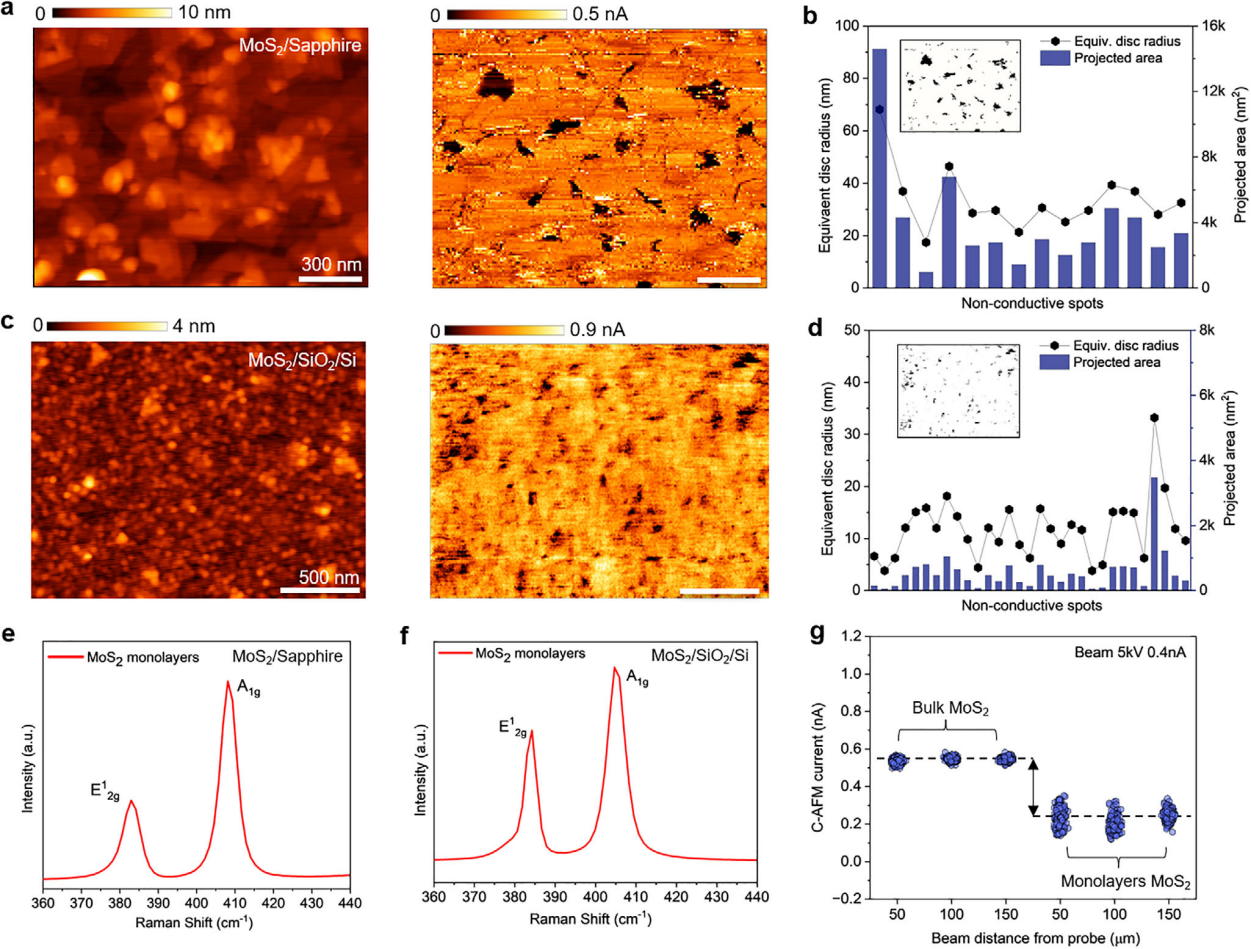
a) AFM topography and C‐AFM image of 3‐5 monolayers MoS_2_/Sapphire sample. C‐AFM is acquired by EBC‐AFM technique at V_acc_ = 5 kV, I_beam_ = 0.4 nA, W_D_ = 10 mm, **∠**i = 90°, and distance_P‐B_ = 30 µm. The scale bar is 300 nm. b) Statistical information on major non‐conductive spots extracted from the corresponding C‐AFM of MoS_2_/Sapphire sample. Inset: masked (20%) image showing only the non‐conductive spots in dark black color. c) AFM topography and C‐AFM image of 2‐3 monolayers MoS_2_/SiO_2_(50 nm)/p‐Si sample. C‐AFM is acquired by EBC‐AFM technique at V_acc_ = 5 kV, I_beam_ = 0.4 nA, W_D_ = 10 mm, **∠**i = 90°, and distance_P‐B_ = 50 µm. The scale bar is 500 nm. d) Statistical information on major non‐conductive spots extracted from the corresponding C‐AFM of MoS_2_/SiO_2_/Si sample. Inset: masked (20%) image showing only the non‐conductive spots in dark black color. e,f) Raman spectra of the MoS_2_/Sapphire and MoS_2_/SiO_2_/Si samples, respectively (taken with a 512 nm laser), showing the two major vibrational peaks of E^1^_2g_ and A_1g_. g) Comparison of C‐AFM current obtained from a bulk (75‐80 layers) MoS_2_ and 3‐5 monolayers MoS_2_ samples through EBC‐AFM technique. Both samples have similar structures of MoS_2_/SiO_2_/Si and applied electron beam is V_acc_ = 5 kV, I_beam_ = 0.4 nA, W_D_ = 10 mm, **∠**i = 90°. The data is recorded by point contact based current versus time (I‐T) spectroscopy with the same probe and same tool in the same day.

Next, we considered two different MoS_2_ samples to perform complete C‐AFM scans using our EBC‐AFM technique and compare them. As shown in the topography images of **Figure** **4**a,c, the first MoS_2_ sample is grown by vapor phase reaction on Sapphire substrate and the second MoS_2_ sample is grown by sulfurization method on SiO_2_/Si substrate, both using the AP‐CVD technique. Further growth details are available in the experimental section. In Figure 4a, the C‐AFM image of the MoS_2_/Sapphire sample obtained at V_acc_ = 5 kV and I_beam_ = 0.4 nA provides important details such as non‐conductive spots, grain boundaries, etc. The non‐conductive spots can be resulted from the uncovered region, point defects, and extended defects, etc. on the MoS_2_ surface. After masking the C‐AFM image with 20% thresholding for noise removal, we extract the equivalent disc radius (R_eqv. disc_) and projected area (A_proj_) of the major non‐conductive spots as plotted in Figure 4b. It is observed that the R_eqv. disc_ of most of the non‐conductive spots falls between 10 and 40 nm, while the corresponding projected area is within 5000 nm^2^. On the other hand, the C‐AFM image of the MoS_2_/SiO_2_/Si sample obtained by using the same electron beam parameters also shows some non‐conductive spots as indicated by the dark area in Figure 4c. However, the overall current level is 3x higher in MoS_2_/SiO_2_/Si sample than the first one. In addition, the R_eqv. disc_ of the non‐conductive spots tends to be smaller as most of them are less than 20 nm having a projected area within 1000 nm^2^. Afterward, we compare the Raman and X‐ray photoelectron spectroscopy (XPS) data of both samples to investigate the higher current level of the second sample. As presented in Figure 4e,f, the characteristic Raman peaks (captured with 514 nm laser) of the MoS_2_/Sapphire (first sample) are at E^1^_2g_ = 383 cm^−1^ and A_1g_ = 408 cm^−1^ indicating 4‐5 monolayers while the MoS_2_/SiO_2_/Si (second sample) demonstrates the E^1^_2g_ peak at 383.5 cm^−1^ and A_1g_ peak at 405.5 cm^−1^ indicating 2‐3 monolayers only.^[^ ^27^ ^]^ That means the second sample has thinner MoS_2_ although conducting more current than the first sample. At this point, we conduct the XPS measurement on both samples, which elucidates the reason for this conductivity difference. From Figure S9a (Supporting Information), we notice that Mo 3d_3/2_, Mo 3d_5/2_, S 2p_1/2_, and S 2p_3/2_ XPS peaks appear at the binding energy positions of 233.8 eV, 230.6 eV, 164.3 eV, and 163.2 eV, respectively, which confirm the semiconducting (2H) phase of MoS_2_ in the first sample.^[^ ^44^ ^]^ In contrast, Mo 3d_3/2_ and Mo 3d_5/2_ peaks in the second sample have been shifted to lower binding energy by ≈1 eV as found in the XPS spectra (Figure S9b, Supporting Information), suggesting the coexistence of metallic (1T) phase MoS_2_ along with the 2H phase.^[^ ^44^, ^45^ ^]^ Furthermore, the fitted XPS spectra indicate the emergence of new peaks (red color) at 230.7 and 228.5 eV that can be attributed to Mo 1T phase while the other peaks at 163.7 and 162.7 eV can be attributed to S 1T phase.^[^ ^46^, ^47^ ^]^ It is worth mentioning here that the lower binding energy of the 1T phase in the XPS spectra is due to the metastability and octahedral coordination of this phase, coupled with a relaxation energy that is lower than that of the thermodynamically stable 2H phase.^[^ ^45^ ^]^ Finally, we conclude that the comparatively higher C‐AFM current level in the second sample (MoS_2_/SiO_2_/Si) is because of the presence of a mixed‐phase in the MoS_2_.

Then we compare between bulk (75‐80 MLs) and monolayers (3‐5 MLs) MoS_2_ samples in terms of C‐AFM current acquired by point contact based I‐T spectroscopy with the same probe and same parameters. Keeping the electron beam at V_acc_ = 5 kV and I_beam_ = 0.4 nA as constant, the current data are recorded for both samples at three different beam distances (50, 100, 150 µm) from the probe. As plotted in Figure 4g, the bulk MoS_2_ demonstrates a steady current level of 0.55 ± 0.01 nA with little variation. However, the monolayers MoS_2_ conducts only 0.25 ± 0.05 nA C‐AFM current, which is almost half of the bulk and has a large standard deviation. This effect can be explained by considering the effective cross‐section area of current collection in the case of bulk versus monolayer. In essence, a larger fraction of injected electrons can reach our probe for the bulk MoS_2_ sample, as they will not be dispersed in the insulating substrate. As a consequence, larger variation of current can be expected in the monolayer sample, as the electronic transport will be dominated by an atomically thick cross‐section area existing between the injected electrons and the probe. At the same time, Figure 4g suggests that the EBC‐AFM technique is well sensitive to bulk and a few monolayers of 2D material samples, given the clearly detectable S/N ratio obtained. Finally, we recognize that various secondary order effects such as lower bandgap, additional interlayer conduction paths, reduced surface scattering, better carrier mobility, and higher secondary emission yield, etc. of the bulk sample can have an impact as well.

**Figure 5:**
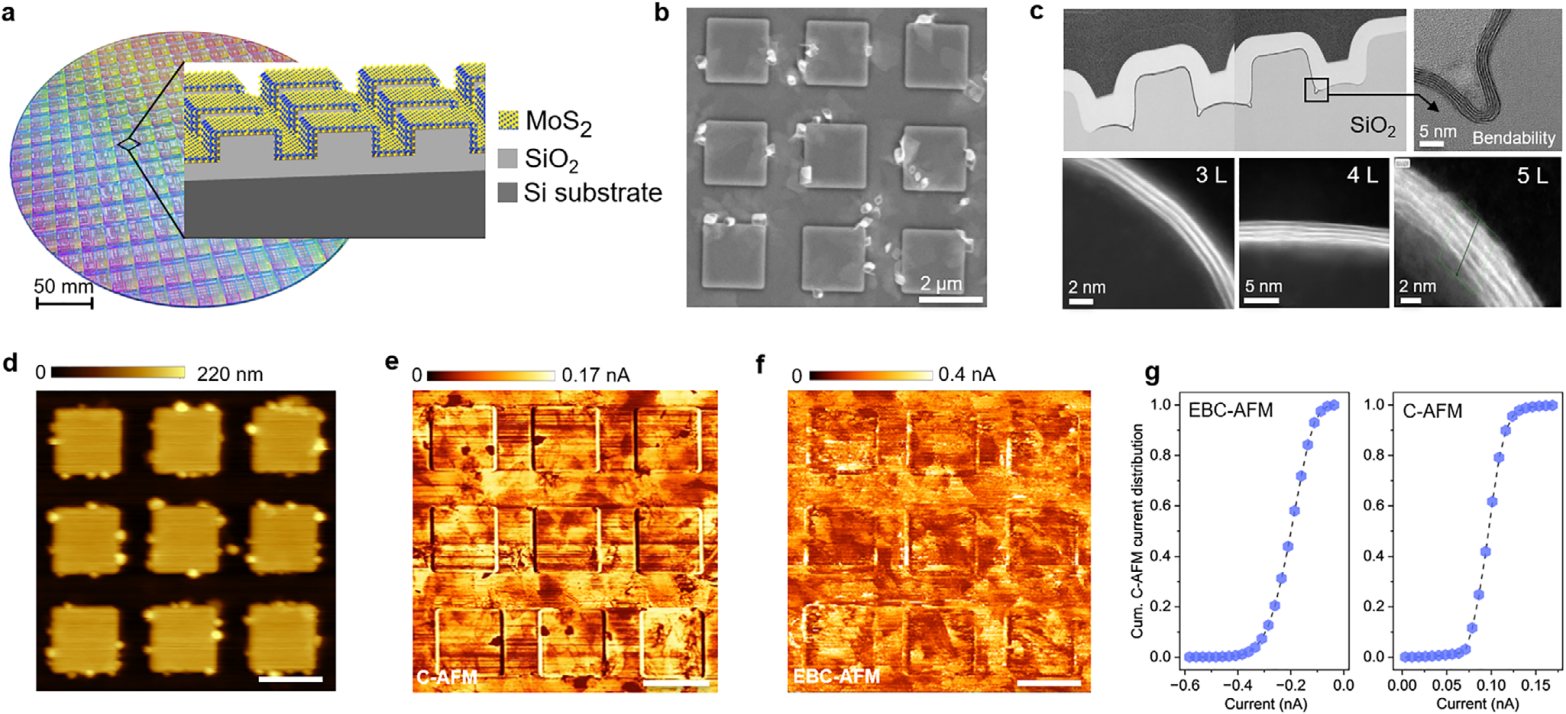
a) A fully processed chip die: schematic of the patterned die where the structure is 3‐5 monolayers MoS_2_/SiO_2_(90 nm)/p‐Si. b) SEM image of 9 × 9 µm^2^ area within the sample. Scale bar is 2 µm. c) Cross‐section TEM images showing the conformal growth of MoS_2_ over the patterned substrate at low and high magnification on SiO_2_. Bottom HAADF‐STEM images show how the flexible MoS_2_ (3‐5 MLs) follow the substrate curvature. d) AFM topography of 9 × 9 µm^2^ area within the sample. Scale bar is 2 µm. e) C‐AFM image obtained by conventional C‐AFM method at 2 V bias with regular conductive probe (tip radius 20 nm). The scale bar is also 2 µm. f) C‐AFM image obtained by EBC‐AFM technique with self‐sensing conductive probe (tip radius 50 nm) at V_acc_ = 5 kV, I_beam_ = 0.4 nA, W_D_ = 10 mm, **∠**i = 90°, and distance_P‐B_ = 30 µm. Scale bar is 2 µm. g) Comparison of cumulative current distribution plots extracted from the EBC‐AFM and conventional C‐AFM images of figure (e,f).

After showing the main features offered by EBC‐AFM and checking its performance on different samples obtained in small dimensions, here we test our methodology on a fully processed chip die as illustrated in **Figure** **5**a, where the MoS_2_ is grown by direct growth method on a target layer, i.e., SiO_2_ patterned in a dense array of squared pads. The minimum thickness of the SiO_2_ is 90 nm, and the pads have a dimension of 2 µm ×2 µm × 0.2 µm, covering the entire surface of the chip die (Figure 5b,d). The Raman spectra of the MoS_2_ is also available in Figure S10 (Supporting Information). During the direct growth of MoS_2_, a Perylene based nucleation promotor is used, which is commonly used for wafer‐scale synthesis of 2D materials with great bendability and flexibility. Further growth details are available in the experimental section. As shown in Figure 5c, the cross‐sectional Transmission electron microscopy (TEM) and High‐angle annular dark‐field scanning transmission electron microscopy (HAADF‐STEM) images clearly show how well the MoS_2_ (3‐5 MLs) can follow the curved geometry due to its extraordinary bendability achieved with the help of the promoter. This example is reported as a direct growth method, which is considered one of the main pathways for large area applications of 2D materials and high‐performance devices. However, direct growth requires high temperatures, thus impacting the quality of crystallinity and conformality in MoS_2_. Therefore, we use a sample to mimic a realistic analysis flow using EBC‐AFM, where our technique can be used to assess the level of conformality, homogeneity, and coverage with an approach that is fully compatible with in‐line wafers metrology. While Figure 5b–d show the structural information of our sample by means of SEM, TEM and AFM (morphology), Figure 5e,f compare the results obtained on the same die, using conventional C‐AFM and EBC‐AFM, respectively. Here, the conventional C‐AFM image is obtained at 2 V bias with regular conductive probe, while the EBC‐AFM is performed with a self‐sensing conductive probe at V_acc_ = 5 kV, I_beam_ = 0.4 nA, W_D_ = 10 mm, **∠**i = 90°, and distance_P‐B_ = 30 µm. Noteworthy, both images show a comparable level of details for the MoS_2_ quality, with visible formation of lowly conductive grains and the appearance of MoS_x_‐clusters on the edges of the pads. According to the levels of current obtained (on average) in Figure 5e,f, we conclude that the electrostatic of tip‐sample junction is comparable between the two experiments. The cumulative current distributions (Figure 5g) extracted from both C‐AFM maps are also in good agreement. Here, we emphasize that the gap in S/N visible for the EBC‐AFM when compared with a standard stand‐alone C‐AFM instrument is likely to be ascribed to the inferior sensing performance of the compact self‐sensing probe system used in our experiment and not to any intrinsic limitation of probing physics. A real‐time video of data acquisition and visualization by EBC‐AFM method is available in Video S1 (Supporting Information). Clearly, the major value of the result is the proof of the feasibility of EBC‐AFM for a fully integrated characterization flow, for example, customizing existing e‐beam inspection instruments with the addition of a high‐performance AFM system, thus capable of executing electrical modes without back‐contacts over the entire wafer area.

Over and above that, we compare the maximum achievable current level using the conventional C‐AFM method with respect to EBC‐AFM on the same MoS_2_ sample. It is due to the fact that conventional C‐AFM tools usually can apply a maximum of ±10 V bias; however, sometimes this bias may not be adequate in the case of extremely resistive 2D materials. As visible in Figure S11 (Supporting Information), the current map obtained by conventional C‐AFM at 10 V manifests the highest current level within 4 nA, whereas a similar current level is attained just by applying I_beam_ = 6.3 nA through the EBC‐AFM. It is noted that if required, 50–100 nA high beam current can be easily applied by SEM. Therefore, our EBC‐AFM can eliminate the dependency of limited voltage supply for charge carrier injection to the sample, as conventional C‐AFM requires. Furthermore, the ambiguity that arose from the voltage polarity in conventional C‐AFM imaging of the 2D materials^[^ ^28^ ^]^ (due to bulk‐limited/injection‐limited carrier transportation) is no longer applicable in EBC‐AFM imaging because of its direct carrier injection ability to the sample. What's more, since the EBC‐AFM technique utilizes an AFM‐in‐SEM module to carry out the experiments, it enables concurrent evaluation of the 2D material surface to avoid any scratching on it due to high tip‐sample contact force and assessment of the tip‐apex condition, as pointed out in Figure S12 (Supporting Information). Thus, our EBC‐AFM technique outperforms the conventional C‐AFM in many ways as discussed above, and breaks the barrier of limitations.

## 3 Conclusion

In this article, we have reported the development of a characterization method for 2D materials that breaks free from classic limits of contact‐mode electrical AFMs, enabling high‐resolution, nm‐precise electrical characterization of 2D materials, at wafer scale and without the need for interruption of the chip manufacturing process flow. We demonstrate all the functionalities of standard metallic back‐contacts on the sample surface, here replaced with an e‐beam illumination that can be easily located in the area of interest, with no physical intervention on the sample surface. We have presented the main technical description and hardware implementation of the technique while demonstrating its application to various types of MoS_2_ grown with multiple methods to prove the applicability of our approach to a wide range of sensing configurations. Second, we have then focused on the main parameters of the e‐beam illumination that impact the quality of the electrical channel of the AFM mode. Here, we worked on establishing a set of operations to maximize the S/N ratio probed at the tip‐sample junction, thus improving the quality of our measurements, to the point that our EBC‐AFM can be considered as interchangeable with a conventional lab‐scale C‐AFM operation. Finally, we demonstrated the optimization of the method to a real case study for the surface of fully fabricated patterned chips, where a thick insulating layer is covered by MoS_2_ deposited through a direct growth method. Here, the relevance of these material systems relies on their compatibility with the CMOS manufacturing process, demonstrating how our solution can be directly translated into a large area, full‐wafer analysis flow, which up until now has not been possible due to process contamination and the need for breaking the wafer into small coupons. Our results show a clear pathway to leverage e‐beam illumination, widely applied as in‐line defect monitoring, for the execution of electrical AFMs. This allows for the integration of a new analysis flow to access to a wide range of materials properties in 2D materials, including homogeneity, local defects density, crystallinity, and electrically active defects down to single digit nm precision. Given the large attention of the community to the full‐scale integration of 2D materials‐based devices, our work paves the way to the lab‐to‐fab transition for advanced metrology that can close the gap in creating high‐quality atomically thin devices with high‐volume manufacturing.

## 4 Experimental Section

NenoVision LiteScope AFM was used to perform all EBC‐AFM experiments in Nova‐200 and Helios 5 UX SEMs. Pt coated self‐sensing probes were used for Litescope. Traditional C‐AFM and topography of monolayers MoS_2_ samples were done in Bruker Multimode‐8 and Dimension‐3100 AFMs using Pt coated regular probes, i.e., SPARK 70 Pt. Monte Carlo simulation of electron trajectories was done by CASINO v2.5.1 software.

Raman spectroscopies were performed on two different Renishaw InVia spectrometers having 488 and 514 nm lasers, respectively at room temperature. X‐ray photoelectron spectroscopy (XPS) measurements were acquired on a PHI 5600 instrument equipped with a monochromatic Al Kα X‐ray source with an energy of 1486.6 eV and a concentric hemispherical analyzer. The spectra were collected at a take‐off angle of 45° and band‐pass energy of 58.70 eV. The instrument resolution was 0.5 eV. The spectra were aligned using C 1s (285 eV) as a reference. The TEM imaging was carried out by Themis Z G3 aberration‐corrected scanning transmission electron microscope from Thermo Fisher Scientific.

Bulk MoS_2_ crystals were grown via chemical‐vapor transport and exfoliated by first thinning the crystal via the scotch tape method, then using PDMS to stamp the exfoliated crystal onto SiO_2_/Si. SiO_2_ thickness was 300 nm, and SiO_2_/Si substrate was cleaned by sonicating in acetone, ethanol, and IPA for 5 min and dried with N_2_ before the exfoliated MoS_2_ transfer.

Monolayers MoS_2_/Sapphire sample was grown by MoO_3_ and S powder precursors from Vapor phase reaction using the AP‐CVD method. The temperatures used for MoO_3_ and S precursors and the Ar flux during the growth process were 750 and 300 °C with a heating rate of 5 °C per minute, respectively. A detailed growth procedure was reported elsewhere.^[^ ^48^, ^49^ ^]^ The patterned sample having monolayers MoS_2_/SiO_2_(90 nm)/p‐Si was also grown by a similar method, although in the presence of a seeding promoter called Perylene‐3,4,9,10‐tetracarboxylic acid tetra potassium salt (PTAS).^[^ ^49^, ^50^ ^]^ The purpose of the PTAS promoter was to provide appropriate adhesion, flexibility, and bendability of MoS_2_ while growing on a nanopatterned substrate.

On the other hand, few monolayers of MoS_2_/SiO_2_(50 nm)/p‐Si sample (mixed phase) was grown by sulfurization of ammonium heptamolybdate (AHM) based Mo film. The AHM solution, as molybdenum source, was prepared along with the use of an inorganic seed promoter such as Potassium iodide (KI). Further details are available here in a previous publication.^[^ ^51^, ^52^ ^]^

## Conflict of Interest

The authors declare no conflict of interest.

## Supporting information



Supporting Information

Supplemental Video 1

## Data Availability

The data that support the findings of this study are available from the corresponding author upon reasonable request.
