# Supplementary material for: Electron‐Beam Excited Conductive Atomic Force Microscopy for Back Contact Free, Wafer‐Scale and In‐Line Compatible Electrical Characterization of 2D Materials
Source: Adv Sci (Weinh). 2025 Aug 27;12(44):e05113. doi: 10.1002/advs.202505113 (PMC12667532; doi:10.1002/advs.202505113)
Supplement: Supplementary file 1 — Supporting Information [file ADVS-12-e05113-s002.docx]

Supporting Information

**Electron-Beam Excited Conductive Atomic Force Microscopy for Back Contact Free, Wafer-Scale and In-Line Compatible Electrical Characterization of 2D Materials**

Md Ashiqur Rahman Laskar^1^, Sakib Ahmed^1^, Pinakapani Tummala^2^, Alessandro Molle^2^, Alessio Lamperti^2^, Renee Sailus^3^, Youssry Y Botros^4^, Milan Pesic^4^, Rob Davenport^4^, Ondřej Novotný^5^, Jan Neuman^5^, Fabrizio Toia^6^, Ivan Sanchez Esqueda^1^, Seth Ariel Tongay^3^ and Umberto Celano^1,^*

^1^ School of Electrical, Computer, and Energy Engineering, Arizona State University, Tempe, AZ, 85281, USA

^2^ CNR IMM, Unit of Agrate Brianza, via C. Olivetti 2, Agrate Brianza, 20864, Italy

^3^ School for Matter, Transport and Energy Engineering, Arizona State University, Tempe, 85287, AZ, USA

^4^ Applied Materials Inc., Santa Clara, CA, 95054, USA

^5^ NenoVision s. r. o., Brno, 61200, Czech Republic

^6^ STMicroelectronics, Via C. Olivetti 2, Agrate Brianza, 20864, Italy

*Corresponding author: Umberto Celano, Email: umberto.celano@asu.edu

Keywords: Conductive AFM, 2D materials, MoS_2_, wafer compatible, electrical characterization, in-line metrology.


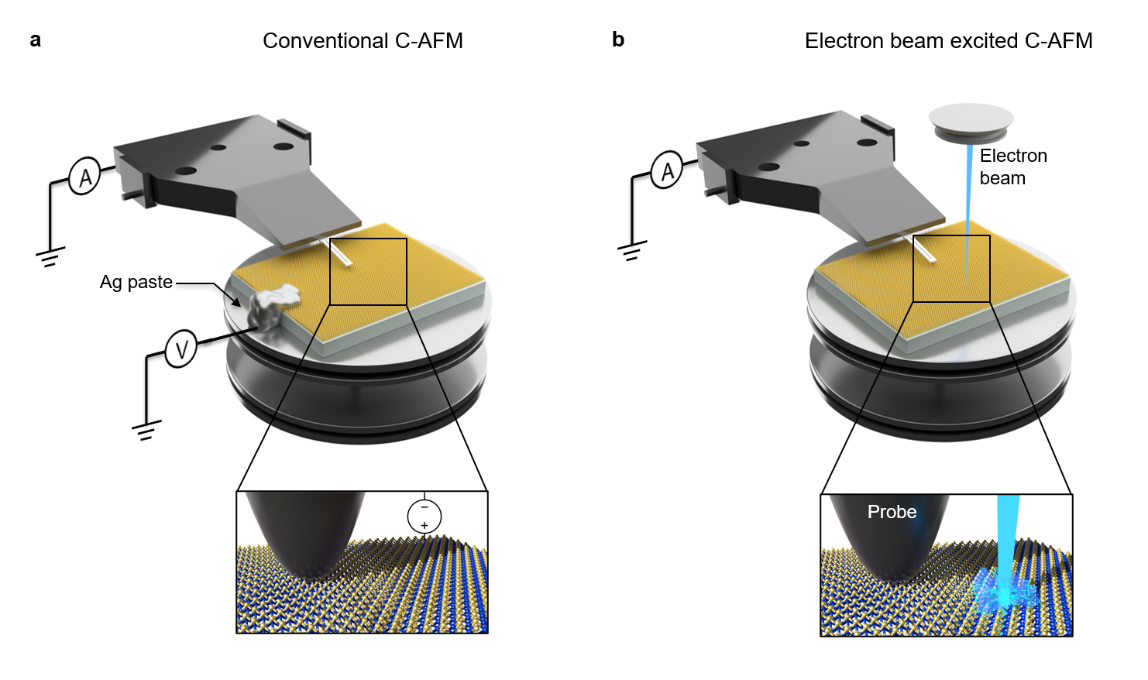


**Figure S1:** **a-b**. Schematics showing the fundamental difference between conventional C-AFM and electron beam excited C-AFM (EBC-AFM) setup.


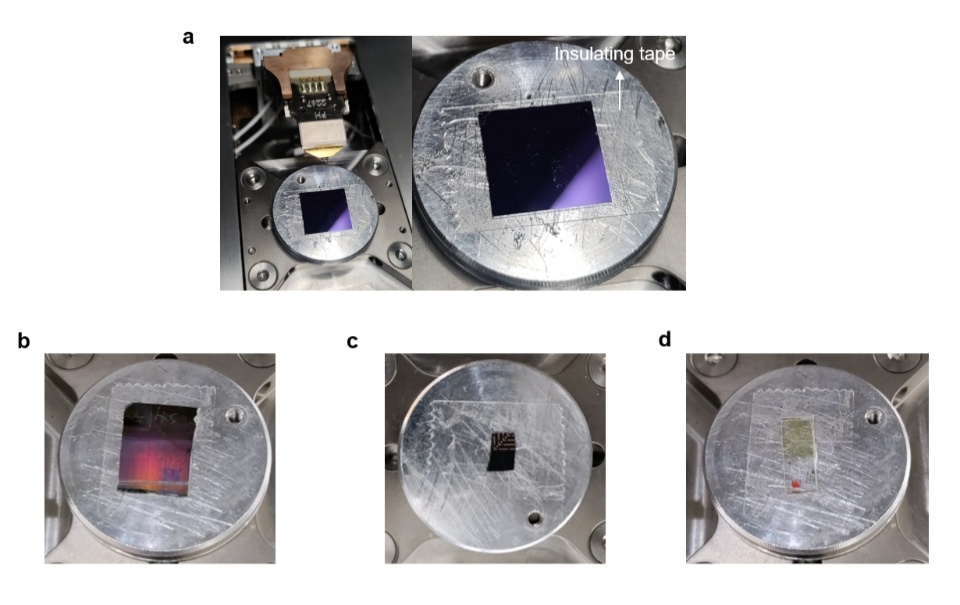


**Figure S2:** Different coupon samples used in EBC-AFM experiments showing how they are mounted to the chuck and scanning module of Litescope AFM with a 50 µm thick double sided insulating tape: **a**. exfoliated MoS_2_ island flakes on SiO_2_/Si substrate **b**. 3-5 monolayers MoS_2_ grown on a patterned SiO_2_/Si substrate **c**. Cu wire on insulating compound mold **d**. 3-5 monolayers MoS_2_ grown on double side polished Sapphire substrate.


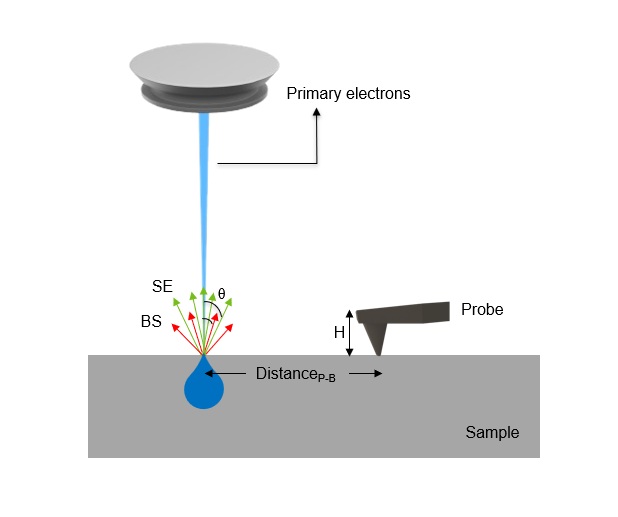


**Figure S3:** A schematic showing the electron beam is impinging on the sample while the conductive probe is scanning at Distance_P-B_. Trivial height (H) of the probes is 8-10 µm that we used in EBC-AFM experiments. The blue color is for primary (incident) electrons and tentative electron trajectories under the sample surface. The green and red arrows are indicating the escaping SE and BS electrons respectively from the surface.


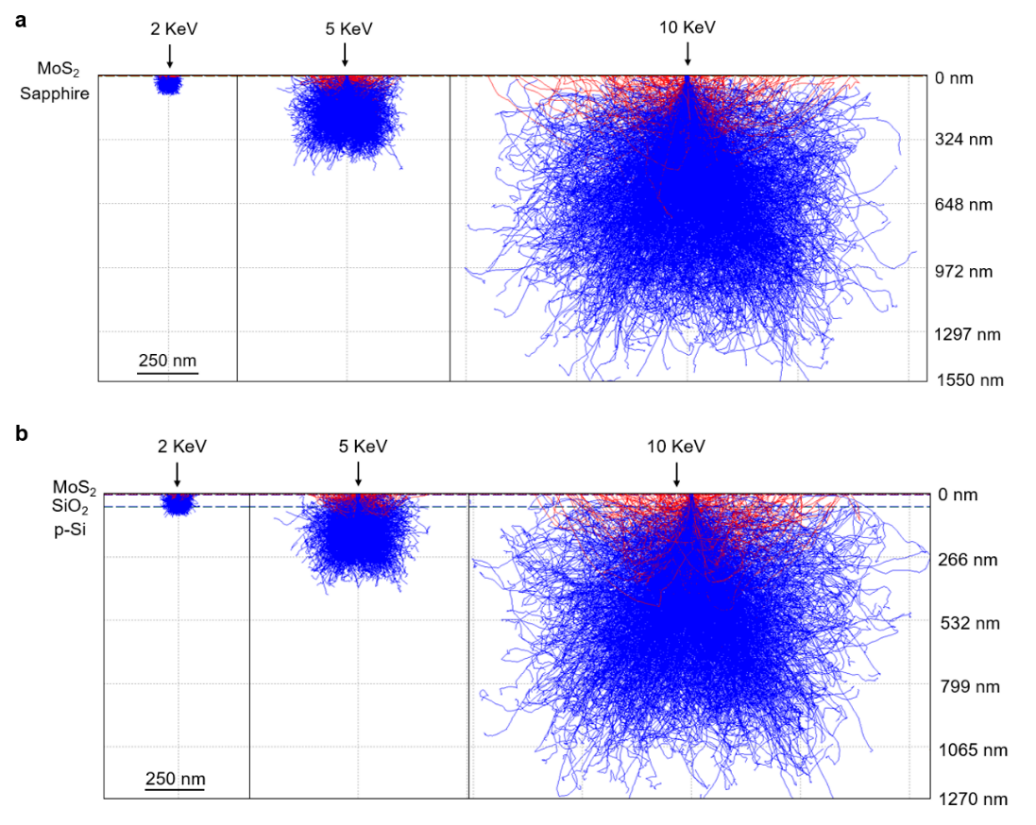


**Figure S4:** Monte Carlo simulation of electron trajectories in **a**. 4-monolayers MoS_2_/Sapphire and **b**. 4-monolayers MoS_2_/SiO_2_/p-Si samples at three different acceleration voltages (2, 5 and 10 keV or kV) of the electron beam. The blue color is for primary (incident) electron and red color is for back-scattered electron trajectories.


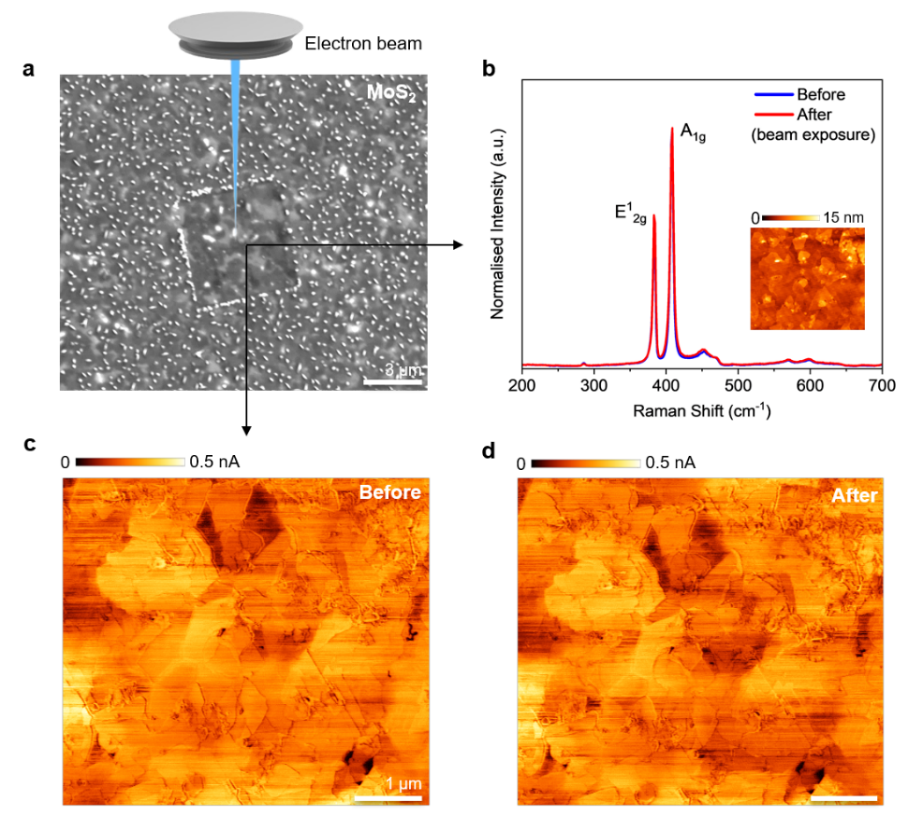


**Figure S5:** **a**. Large area SEM image of a 3-5 monolayers MoS_2_/Sapphire sample showing a comparatively cleaned square size scan area. This area undergoes electron beam irradiation for 15 minutes at V_acc_=5kV and I_beam_=1.6 nA in spot mode. Scale bar is 3 µm. **b**. Raman spectroscopies of the corresponding area before and after electron beam irradiation. Inset: AFM topography of the area. **c-d**. Classic C-AFM images of that area captured by conventional method at 3V bias before and after electron beam irradiation respectively. Scale bar is 1 µm.


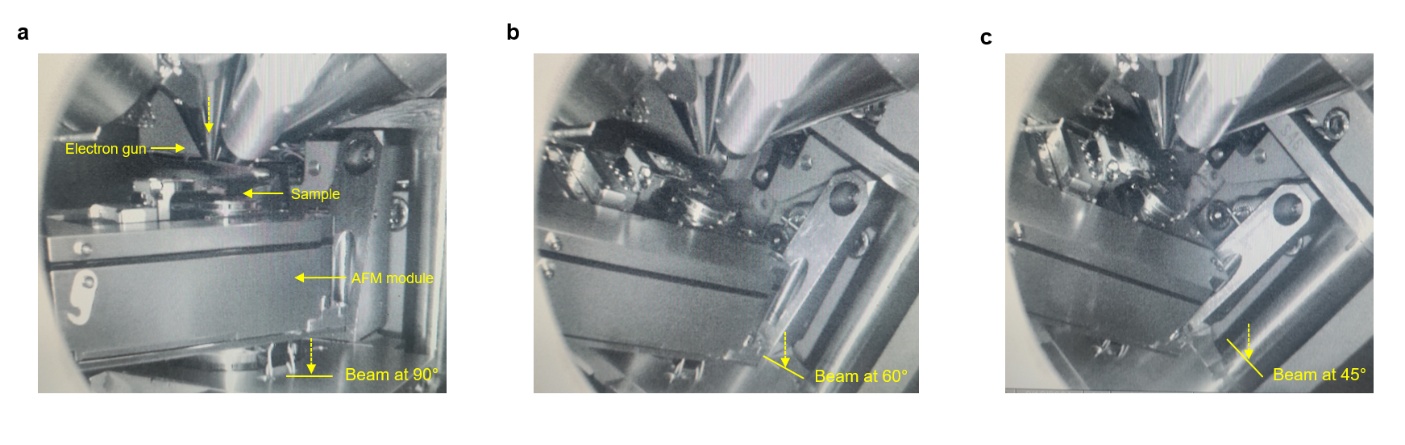


**Figure S6:** **a-c**. Optical photographs of the Litescope AFM inside Nova-200 SEM chamber in vacuum where the AFM module is at different angles with the help of conventional SEM stage movement. Thus, it facilitates the incident electron beam to shine at the sample surface from different angles such as 90°, 60°, 45° etc.


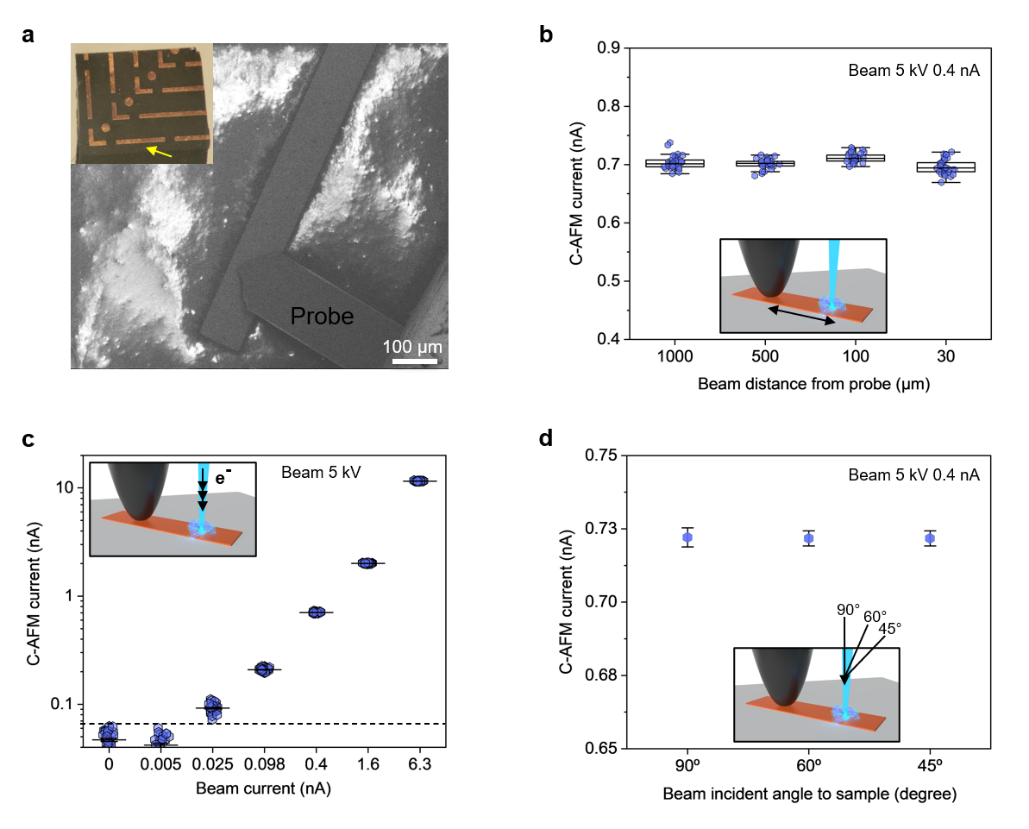


**Figure S7:** **a**. SEM image showing the landed C-AFM probe and Cu wire on insulating compound mold. Scale bar is 100 µm. Inset: optical photograph of the Cu wire sample. **b**. Plot of C-AFM current vs electron beam distance from the probe while illuminating beam at V_acc_=5kV, I_beam_=0.4 nA, W_D_=10 mm, **∠**i=90° on Cu wire. **c**. C-AFM current vs beam current plot when the beam is at distance_P-B_=100 µm and V_acc_=5kV, W_D_=10 mm and **∠**i=90° on Cu wire. The dash line indicates the noise floor of the trans-impedance amplifier of the Litescope C-AFM sensor. **d**. Plot showing the C-AFM current with respect to three different beam incident angles to on Cu wire when V_acc_=5kV, I_beam_=0.4 nA, W_D_=10 mm and distance_P-B_=100 µm. All the data are collected through point contact based current vs time (I-T) spectroscopies.


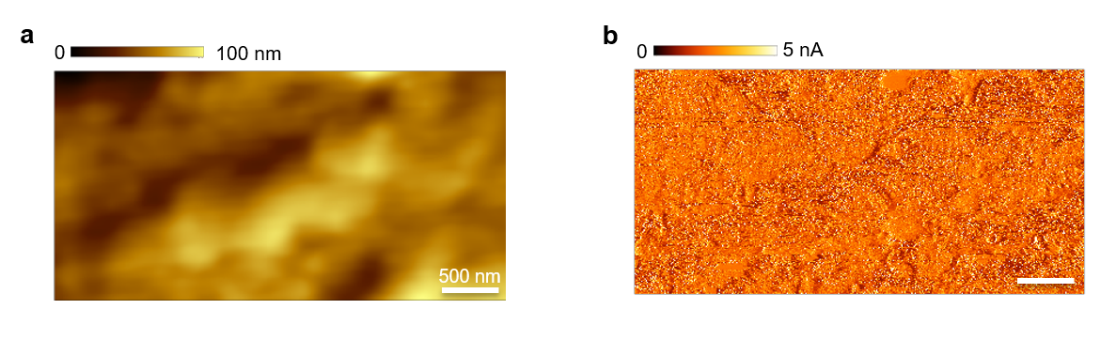


**Figure S8:** **a-b**. AFM Topography and corresponding C-AFM image respectively of the Cu wire sample obtained by EBC-AFM at V_acc_=5kV, I_beam_=1.6 nA, W_D_=10 mm, **∠**i=90° and distance_P-B_=100 µm. The scale bar is 500 nm.


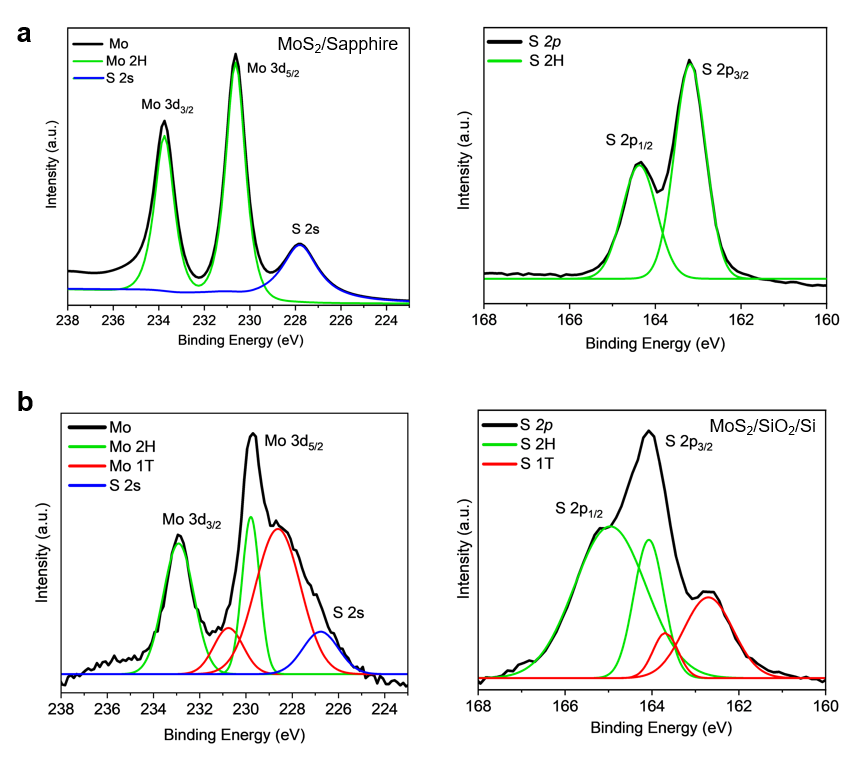


**Figure S9:** X-ray photoelectron spectroscopy (XPS) fitted data highlighting the Mo 3d and S 2p peaks for **a**. MoS_2_/Sapphire and **b**. MoS_2_/SiO_2_/Si samples respectively.


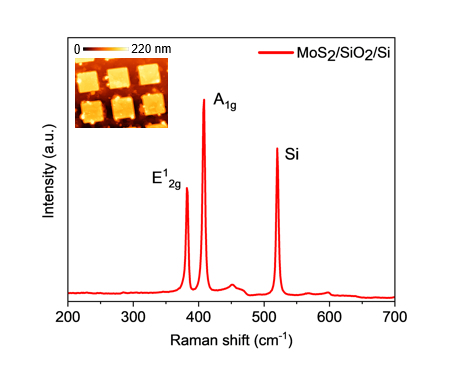


**Figure S10:** Raman spectroscopy of 3-5 monolayers MoS_2_ grown on a patterned SiO_2_/Si substrate. The in-plane and out-of-plane vibrational peaks of MoS_2_ are indicated by E^1^_2g_ and A_1g_ respectively.


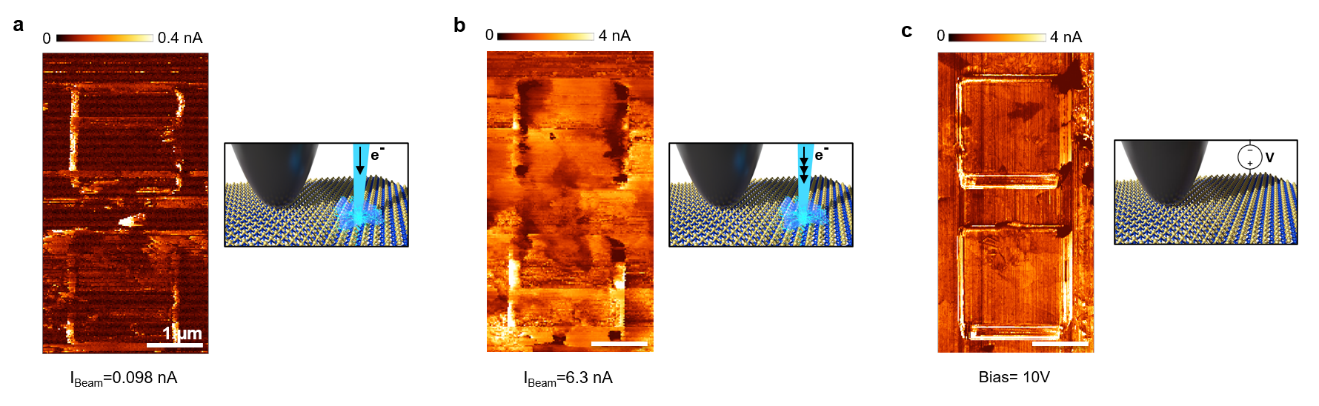


**Figure S11:** **a-b**. C-AFM images of 3-5 monolayers MoS_2_ grown on a patterned SiO_2_/Si substrate obtained by EBC-AFM technique at I_beam_=0.098 nA and I_beam_=6.3 nA respectively with self-sensing Pt coated conductive probe. All other parameters remain constant such as V_acc_=5kV, W_D_=10 mm, **∠**i=90° and distance_P-B_=30 µm. Scale bar is 1 µm. **c**. C-AFM image of the same MoS_2_ sample obtained by conventional method at 10V bias with Pt coated regular conductive probe. The scale bar is also 1 µm.


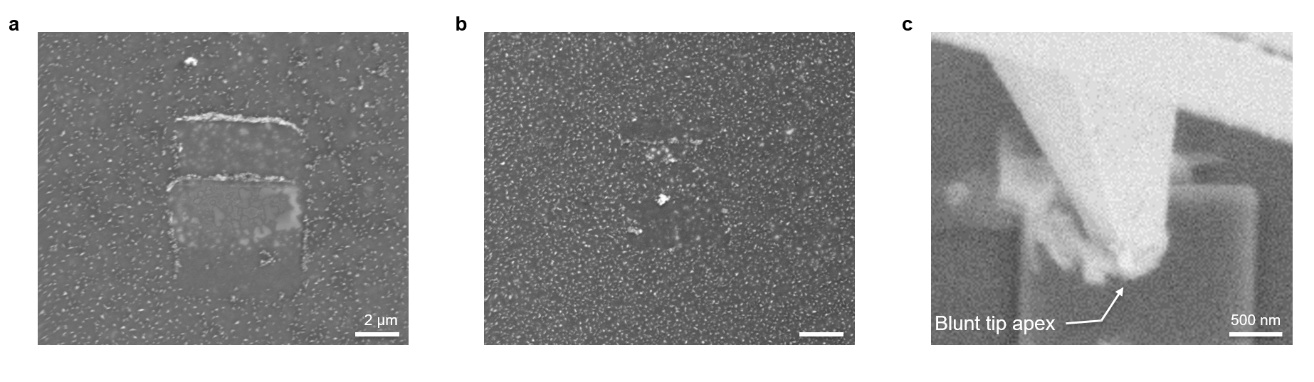


**Figure S12:** **a-b**. SEM images while observing the condition of 2D material sample surface during C-AFM experiments. Scale bar is 2 µm. **c**. SEM image showing the blunt condition of tip-apex of a conductive probe during an experiment. The scale bar is 500 nm.
